# Manually controlled steerable needle for MRI-guided percutaneous interventions

**DOI:** 10.1007/s11517-016-1490-0

**Published:** 2016-04-23

**Authors:** Kirsten R. Henken, Peter R. Seevinck, Jenny Dankelman, John J. van den Dobbelsteen

**Affiliations:** 1 TUDelft, Biomechanical Engineering, Mekelweg 2, 2628 CD Delft, The Netherlands; 2 Imaging Division, Image Sciences Institute, University Medical Center, Heidelberglaan 100, 3584 CX Utrecht, The Netherlands

**Keywords:** MRI-guided interventions, Needle steering, In vitro, Needle deflection, MRI compatible

## Abstract

This study aims to develop and evaluate a manually controlled steerable needle that is compatible with and visible on MRI to facilitate full intra-procedural control and accurate navigation in percutaneous interventions. The steerable needle has a working channel that provides a lumen to a cutting stylet or a therapeutic instrument. A steering mechanism based on cable-operated compliant elements is integrated in the working channel. The needle can be steered by adjusting the orientation of the needle tip through manipulation of the handle. The steering mechanism is evaluated by recording needle deflection at constant steering angles. A steering angle of 20.3° results in a deflection of 9.1–13.3 mm in gelatin and 4.6–18.9 mm in porcine liver tissue at an insertion depth of 60 mm. Additionally, the possibility to control the needle path under MRI guidance is evaluated in a gelatin phantom. The needle can be steered to targets at different locations while starting from the same initial position and orientation under MRI guidance with generally available sequences. The steerable needle offers flexibility to the physician in control and choice of the needle path when navigating the needle toward the target position, which allows for optimization of individual treatment and may increase target accuracy.

## Introduction

Liver cancer is the fifth most commonly diagnosed cancer and the second most deadly form of cancer in men worldwide [11]. In addition to the preferred treatment options (surgical resection and transplantation [7]), multiple other therapies are suggested including high-dose-rate (HDR) brachytherapy. HDR brachytherapy as a therapy for liver cancer is still in an experimental phase [15, 16], but its effectiveness has been shown previously in prostate cancer [14, 24]. HDR brachytherapy is not influenced by the heat losses through blood flow like ablative techniques and is more flexible regarding the number of tumors and the tumor size, so this treatment could be beneficial for patients that are otherwise untreatable.

The success of HDR brachytherapy and other percutaneous needle interventions is highly dependent on accurate positioning of the needle tip. However, this can be complicated by unpredictable needle deflection and target movement due to tissue deformation and breathing [1]. Source placement in brachytherapy is most commonly guided by ultrasound or CT [15, 17], but nowadays interest in MRI is increasing because of its lack of ionizing radiation and its excellent soft tissue contrast [18, 20]. Additionally, MRI can provide structural, functional and physiological tissue parameters, which facilitates immediate visualization of the effect of the treatment [12, 19]. Currently, needles are navigated to the target position by means of an iterative process in which the needle is advanced and imaged subsequently in case of image guidance through CT or fluoroscopy. In interventions guided by ultrasound, needle insertion and imaging are executed simultaneously. Irrespective of the imaging modality, the direction of insertion can hardly be adjusted once the needle has penetrated the tissue, which limits the control options to either advancing or retracting and reinserting the needle.

This research aims at the development of a manually controlled steerable needle that is compatible with and visible on MRI to provide active control of the needle trajectory during insertion for accurate tip positioning. Multiple steering strategies have been proposed previously, which include base manipulation [4, 8], telescopic mechanism [5, 22], and asymmetric (beveled and precurved) needle tips [6, 13, 23]. Most of these approaches involve simultaneous control of multiple degrees of freedom, computer controlled path planning and predictive models, which is not yet attainable for implementation in clinical practice. This work describes the design and the validation of a needle that can be manually actuated and controlled by the physician under image guidance and thereby offers flexibility in navigation to the target position. The performance of the instrument is further evaluated by an in vitro MRI-guided intervention.

## Methods

### Steerable needle

**Fig. 1:**
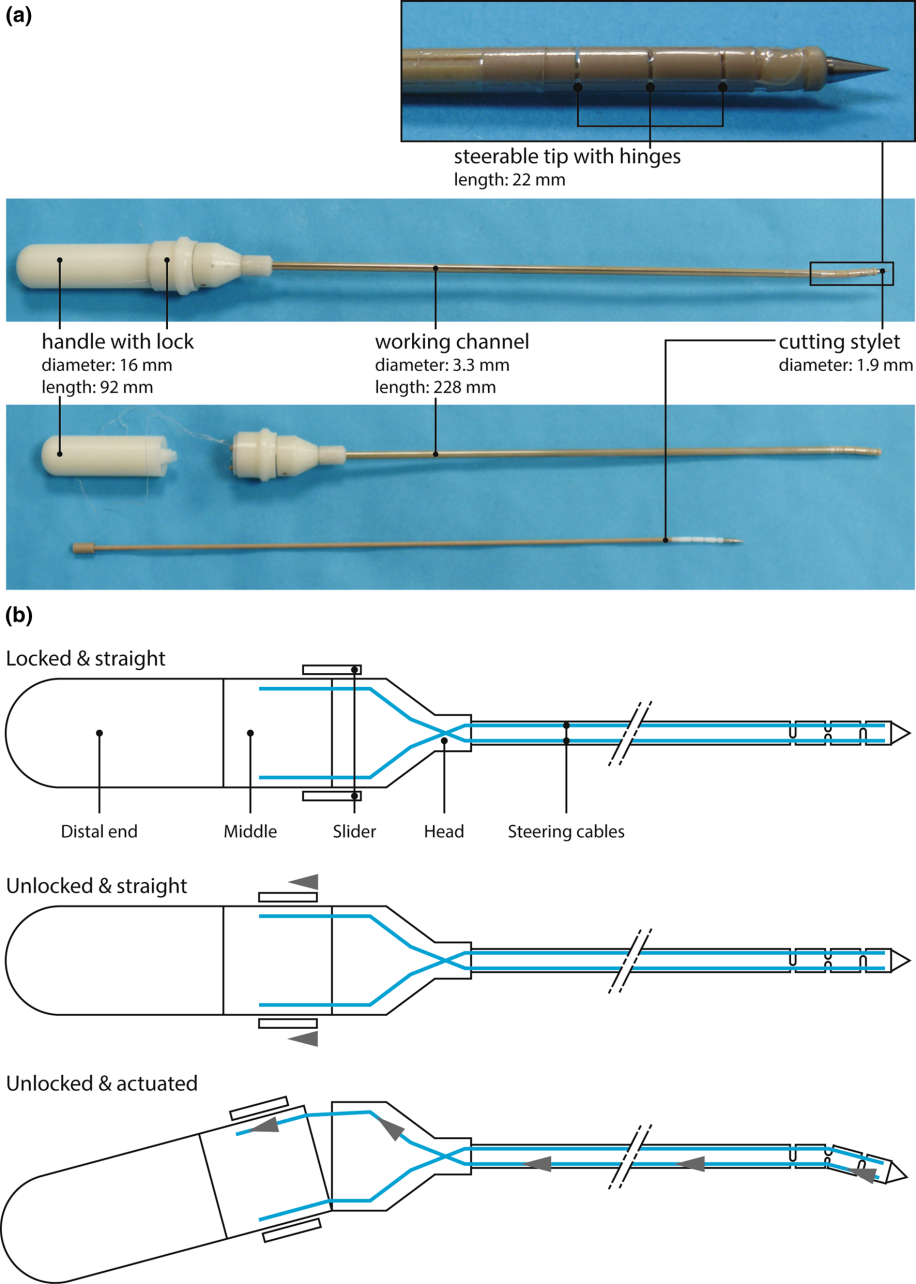
The steerable needle: **a** handle, steerable shaft, and cutting stylet; **b** working principle

The manually controlled steerable needle consists of three main parts (Fig. 1a): cutting stylet, working channel, and handle with slider to lock the steering mechanism. The distal part of the working channel can actively be oriented into the right direction with the handle, while the flexible needle shaft passively follows as the needle progresses through the tissue. The cutting stylet runs through the working channel and can be replaced by a diagnostic or therapeutic instrument when the target has been reached. The working principle of the manually controlled steerable needle is visualized in Fig. 1b. The extremities of four steering cables are attached to the middle part of the handle and to the tip of the working channel, respectively. The distal part of the needle can be locked in a straight position by pushing the slider forward. When the lock is released, the handle can be rotated with respect to the needle, so that at least one of the cables is pulled, which causes the needle to deflect at the distal end. Rotation of the handle is bounded through a mechanical stop in the handle which limits the bending angle at the distal end to 20° in all directions, which is expected to allow for sufficient needle deflection in the clinical application.

The cutting stylet with a diameter of 1.9 mm consists of three parts: a flexible polyether ether ketone (PEEK) tube; a highly flexible part made out of Teflon; and a titanium conical tip connected to each other by means of press fittings. The titanium tip generates a larger artifact than other parts, which enhances its visibility. The working channel is a PEEK tube with an outer and inner diameter of 3.2 and 2.0 mm, respectively. Four grooves are milled along the working channel to provide space for the Dyneema steering cables (Nanofil, Berkeley, USA). In addition, three pairs of radial grooves are milled in the distal part of the working channel to create compliant elements in the steerable tip with a length of 22 mm. The three pairs are positioned with a spacing of 5.0 mm and oriented in 120° with respect to each other to allow for bending of the tip in all directions. The working channel is covered with a biocompatible polyethylene terephthalate (PET) shrinking tube (103-0302, Vention Medical—Advanced Polymers, USA). The polyoxymethylene (POM) and PEEK handle consists of four main parts (Fig. 1a): (1) the distal end that provides an easy pencil grip; (2) the middle part to which the cables are attached; (3) the head of the handle that contains a hinge and connects to the needle; and (4) the slider with which the needle can be fixed in the straight position.

### Validation of steering

**Fig. 2:**
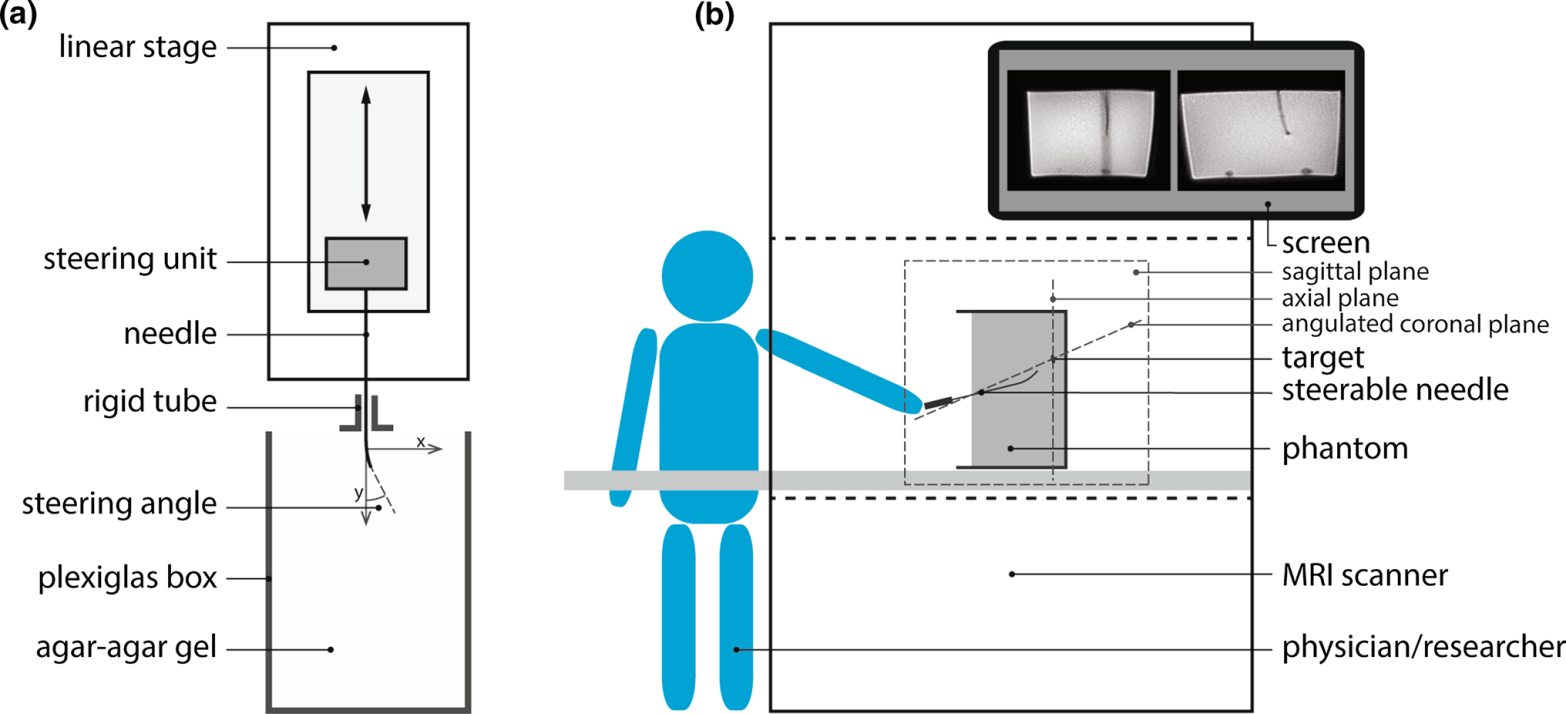
Schematic views of the experimental setups: **a** evaluation of the steering mechanism using a linear stage; **b** evaluation of the integration of the steerable needle in an MRI-guided treatment

The relationship between the steering angle of the tip and the resulting path curvature was evaluated in an in vitro setup (Fig. 2a). The needle was attached to a linear stage (Aerotech PRO 115, Aerotech Inc., USA) and the steering angle at the distal end of the needle was fixed. Then the needle was moved down with a constant speed of 5 mm/s into a gel phantom (1 % Agar–Agar and 0.9 % NaCl in 2 L water), until the needle had reached an insertion depth of approximately 60 mm. During insertion in *y* direction, the needle was registered with an optical camera with a resolution of 0.12 mm. The resulting images were stored for analysis of the deflection of the needle in homogeneous material afterward. Three constant steering angles (4.7°, 12.6°, 20.3°) were applied to the needle tip in *x* direction. After this, the gel phantom was replaced by a tissue phantom that consisted of a porcine liver embedded in gel to gain insight in the steering behavior in inhomogeneous tissue. The optical camera was replaced by a transducer (6C2 Ultrasound Transducer, Siemens) and an ultrasound imaging system (Acuson Sequoia 512, Siemens, Germany) that registered the needle tip at a rate of 25 frames per second with a calibrated accuracy of 0.375 × 0.333 mm. Each experiment was repeated six times per steering angle to gain insight in the variation in deflection while preventing the needle from following the same trajectory twice.

### MRI-guided intervention

The aim of the in vitro MRI-guided intervention was to show how the needle trajectory can be controlled using real-time feedback obtained by MRI. The needle was inserted in a gel phantom (15 % gelatin in 5 L water in a 15 × 17 × 21 cm transparent box) to reach targets at different locations while starting from the same entry point and initial orientation. The targets (olives) were positioned at a depth of 120 mm. The first target was positioned 31 mm to the right and 2 mm below the point of insertion. The second target was positioned 4 mm to the left and 24 mm above the point of insertion. The phantom was positioned in the middle of the bore of the 1.5 T whole-body MRI scanner (Philips Healthcare, The Netherlands), and the open side of the box was aligned with the opening of the bore. A coil setup consisting of two elliptical elements of 14 × 17 cm was applied for signal reception. One of the researchers was positioned next to the scanner and inserted the needle in the phantom to subsequently reach two targets starting at the same point of insertion. Figure 2b provides an overview of the setup of this experiment.

A volume scan was made for planning. Based on the volume scan, angulated coronal and sagittal planes were selected in such a way that both the needle tip and the target were enclosed in the interception of the planes. These planes visualized the deflections of the needle to the left and to the right and deflections upward and downward. The fast dual-plane dynamic scans of these planes were shown on a display in the MRI-room to provide insight in the required steering motion to the researcher during the procedure with an update frequency of approximately 0.5 Hz. The scan parameters of the volume scan and the intra-procedural dual-plane dynamic scans are provided in Table 1. Advancement of the needle was executed iteratively by subsequently updating the dual-plane dynamic scans, deciding on the manipulation required to reach the target (left–right and up–down) and applying the manipulation while advancing the needle approximately 1–2 cm. Both the volume and the dual-plane scans obtained during the procedure were stored for post-procedural analysis to verify whether the targets had been reached.

**Table 1:** MRI scan parameters

	Volume scan	Dual-plane dynamic scan
Type	3D, ultra-short echo time, free induction decay sampling with a center-out radial read-out	2D, free induction decay sampling with a center-out radial read-out [11]
Field of view	192 × 192 × 192 mm^3^	192 × 192 mm^2^
Slice thickness	–	10 mm
Acquired/reconstructed isotropic voxel size	1.5/1.0 mm	1.5/1.0 mm
Echo time (TE)	0.34 ms	0.75 ms
Repetition time (TR)	3.27 ms	3.34 ms
Flip angle	15°	25°
Read-out bandwidth	1332 Hz/pixel	1332 Hz/pixel
Scan duration	1 min 48 s	2.1 s/dynamic scan

The 3D volume scans were post-processed according to a reconstruction method known as center-out radial sampling with off-resonance (coRASOR) reconstruction. This reconstruction aims at selective depiction of the needle with high positive contrast. CoRASOR reconstructions were performed in MATLAB (The MathWorks, USA) using an off-resonance value of 5000 Hz. A detailed description of both the principles and the workflow of the coRASOR reconstruction method can be found elsewhere [2, 3, 21]. Background suppression was obtained by subtracting the on-resonance image from the coRASOR-reconstructed image [21].

## Results

### Validation of steering

**Fig. 3:**
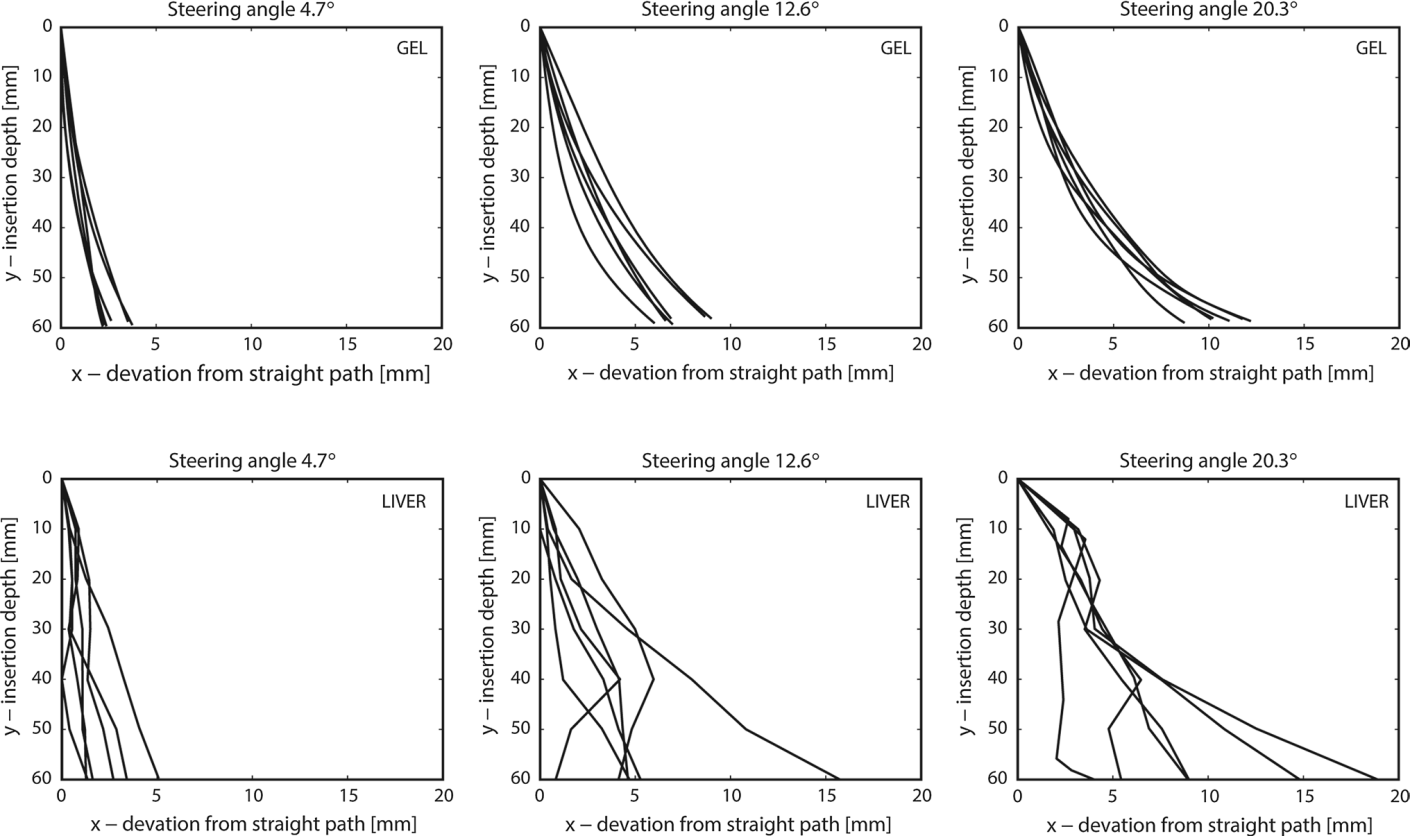
The trajectories that the needle tip followed during insertion in the gel or liver phantom. Generally, a larger steering angle results in more deflection of the needle than a smaller angle

Figure 3 shows the trajectories that the tip of the needle followed during insertion in the gel as monitored by the optical camera. Deflection is defined as the perpendicular distance between the actual needle trajectory and a straight vertical trajectory. As expected, a larger constant steering angle results in more deflection, although variation is present. After the needle was advanced over 60 mm in the gel phantom, the deflections ranged from 2.2–3.8 mm, 6.3–9.6 mm, and 9.1–13.3 mm for a constant steering angle of 4.7°, 12.6°, and 20.3°, respectively. The deflections of the needle at an insertion depth of 60 mm in the liver phantom ranged from 1.3–5.1 mm, 0.8–15.7 mm, and 4.6–18.9 mm for a constant steering angle of 4.7°, 12.6°, and 20.3°, respectively.

### MRI-guided intervention

First the needle was inserted and a volume scan was made to determine the location of the needle tip and the target. Based on this scan, the two imaging planes to be displayed intra-procedurally were selected. An example of the dual-plane dynamic scans is shown in Fig. 3a. These images demonstrate that the needle can clearly be visualized, without inducing large image distortions of signal voids. The first plane was an oblique plane between the coronal plane and the transversal plane. This plane provided information about steering actions in the left–right direction. In this plane, the tip visualization is enhanced through its artifact. The second plane was orthogonal to the first plane and provided information about upward and downward steering. Both planes included the entry point and the target. The hypointense band that is visible in the second plane indicates the cross section of the first imaging plane and is caused by saturation effects present due to the short acquisition times of the dual-plane imaging sequence.

**Fig. 4:**
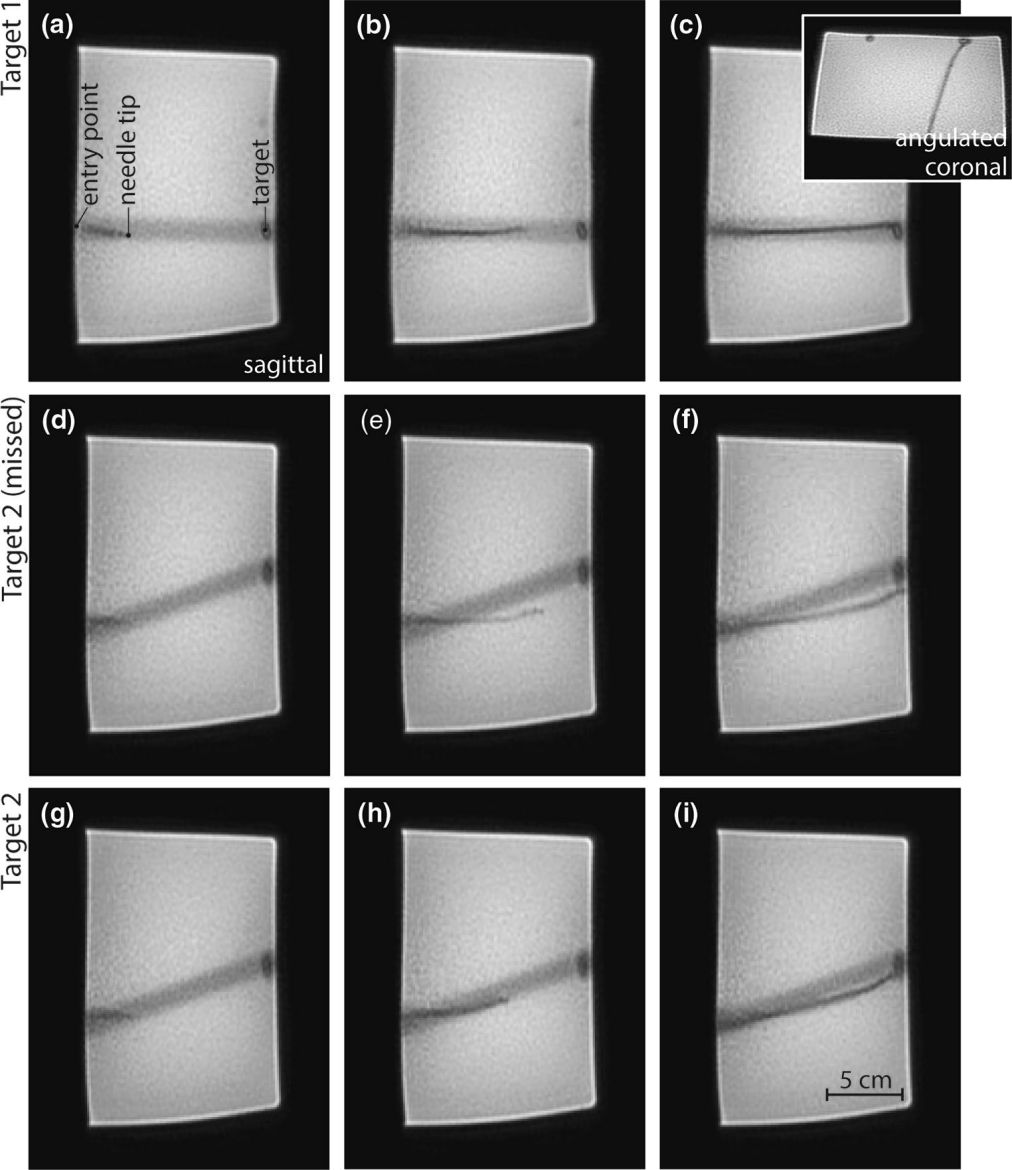
A selection of the two-plane images provided to the researcher during needle insertion: **a**–**c** the iterative process in which the first olive is targeted; **d**–**f** the first attempt to target the second olive; and **g**–**i** the final and successful attempt to target the second olive

Figure 4 provides a selection of dynamic images of the second plane obtained in between the iterative insertion steps to reach the two targets. The first three images (Fig. 4a–c) show three out of the seven steps in which the needle is approaching the first target. After the first target has been reached, the needle is retracted and new planes are selected that include the second target. Figure 4d–i shows six out of eleven steps that were required to target the second olive. The first attempt to target the second olive (Fig. 4d–f) was not successful, because steering was only initiated when the needle tip was already close to the target. Therefore, the needle was withdrawn and the target was reached in a second attempt (Fig. 4g–i).

**Fig. 5:**
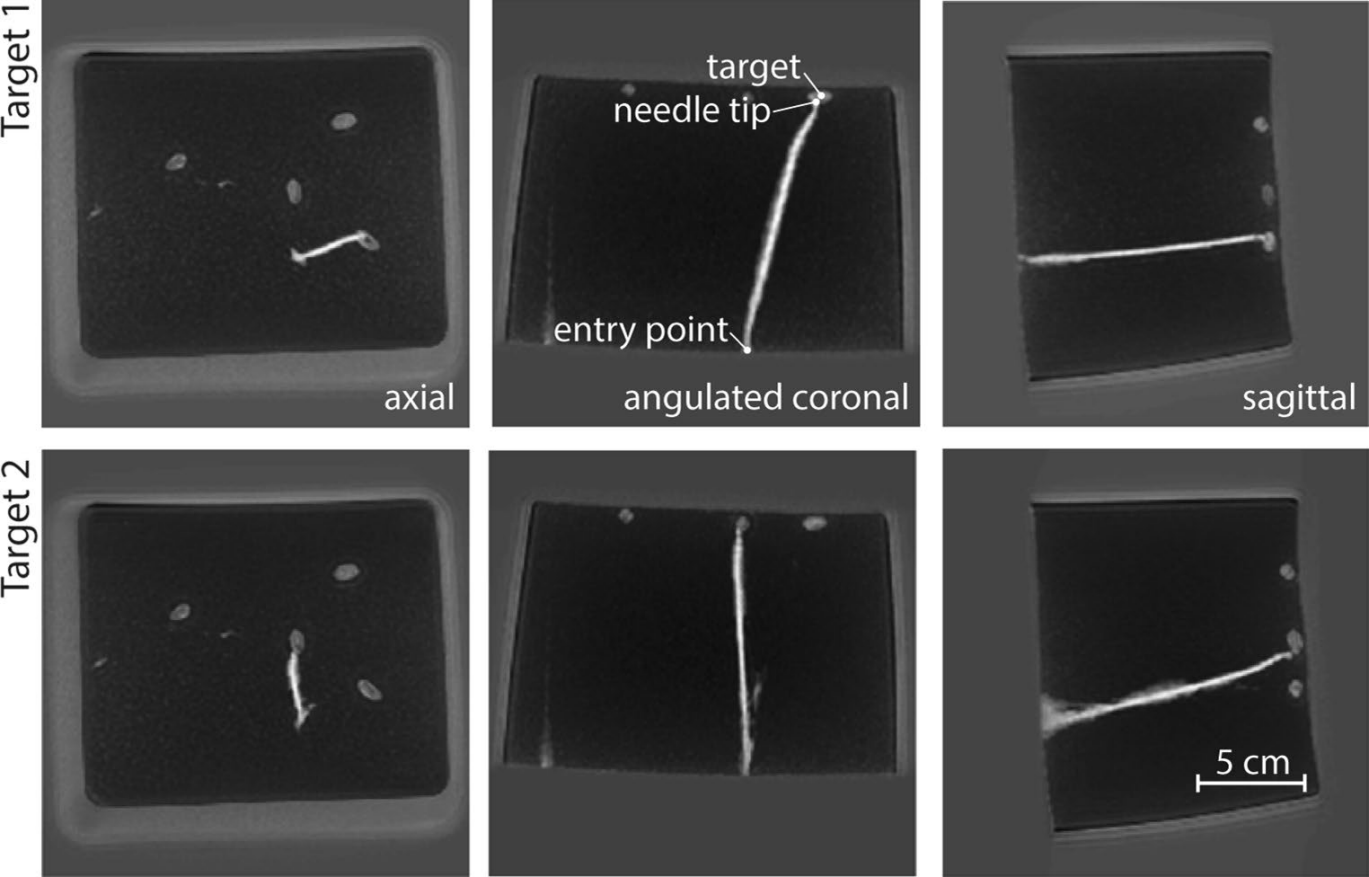
Orthogonal scans of the final needle trajectory obtained through post-processing by means of the coRASOR reconstruction

After the intervention, the MRI scans were subjected to post-processing. CoRASOR-reconstructed 3D volume scans enabled positive contrast visualization of the needle trajectories in 3D, as shown in Fig. 5. The targets are depicted with relatively high intensity as compared to the gel medium on the maximum intensity projections, but the needle is presented hyperintense. The orthogonal maximum intensity projections confirm that both targets are successfully reached from a single entry point while solely supported by 2D MRI guidance.

## Discussion

This work aimed at the development and evaluation of a manually controlled steerable needle to provide active control in MRI-guided interventions that require accurate targeting, such as HDR brachytherapy. The orientation of the tip of the proposed needle could be adjusted by manipulating the handle thereby steering the needle in the required direction. The shaft of the needle passively followed. The needle was designed to be compatible with MRI and mainly consists of plastics. The titanium tip was the only exception and generated an artifact that improved its visibility on MRI.

In vitro experiments in gel showed that the steering angle applied to the tip of the needle determined the degree of deviation from a straight trajectory during insertion, which indicated that the needle trajectory could be adjusted by manipulating the tip orientation. Variations in deflection resulting from the same steering angle at the tip may have been due to variable friction and other mechanical losses in the needle. This did not necessarily affect the target accuracy since the trajectory could be adjusted at any time during insertion. The degree of deflection may be sufficient for the clinical application, but the maximum steering angle can be increased if necessary by adjusting the handle design.

A second set of in vitro experiments was performed to further evaluate the needle in an MRI-guided procedure. In these experiments, the needle was successfully navigated to two targets at different locations starting from the same entry point with the same orientation, illustrating several important findings: (1) the developed needle can be visualized using a generally available dynamic MR imaging technique using a clinically relevant and realistic slice thickness of 10 mm; (2) the needle does not induce large image distortions or signal voids, enabling the simultaneous depiction of both the needle and surrounding soft tissue; (3) the steering abilities of the needle facilitates accurate navigation, enabling MR-guided targeting of predefined locations; (4) the paramagnetic properties of the titanium tip facilitate selective positive contrast visualization when increased specificity is necessary.

Interventional radiologists indicated that the update frequency of 0.5 Hz is sufficient, because needle insertion during the intervention usually takes several minutes. However, a higher update frequency provides more feedback, which may improve the positioning accuracy. Recently we have demonstrated that the update frequency can be increased up to four times by using sliding window reconstruction approaches with compressed sensing reconstruction, leading to update frequencies of 2 Hz for a dual-plane scan technique [25].

A steerable needle offers active control to the physician, so that the trajectory of the needle can be adjusted intra-procedurally. This reduces the need for retraction and reinsertion when the planned trajectory is not resulting in the correct position of the needle tip due to unforeseen needle deflection or tissue deformation. The steerability of the needle also introduces the possibility to reach multiple targets from a single point of insertion, which could be of added value when therapy needs to be applied to multiple locations (e.g., in case of multiple tumors). In addition, curved paths can be obtained so that penetration of critical and vulnerable structures (e.g., blood vessels or the lungs) can be avoided. The proposed needle could therefore facilitate individual treatment optimization and contribute to the accuracy of needle placement in image-guided interventions such as HDR brachytherapy in the liver.

The steering mechanism presented in this work has a number of advantages with respect to previously described steerable needles. In needle steering by means of base manipulation [4, 8], moments are applied around the point of insertion to reorient the needle tip. This can result in large forces around the entry point and along the needle. In the current mechanism, the forces that are required for steering are only initiated at the needle tip. When needle steering is obtained through a telescopic mechanism in which multiple concentric pre-curved tubes protrude through each other [5, 22], extensive pre-operative planning is required to translate the planned trajectory into control inputs that are needed to realize this trajectory. Operation of the current needle is more simple: the orientation of the needle tip can be adjusted at any time during insertion by reorienting the handle in the preferred direction. Operation of other needles that rely on the steering effects of asymmetries at the tip [6, 13, 23] is similar, but additional rotation around the longitudinal axis is required to adjust the direction of steering and to control the degree of steering.

Although the needle provides control of the trajectory, some demerits need to be taken into account. The experiment in the MRI showed that steering becomes more difficult when the needle is inserted deeper. This suggests that timely steering is preferred over last-minute adjustments. In addition, the freedom of movement of the physician is limited by the confined space in the bore of the scanner, which may affect the control options. Finally, applicability may be limited when it is expected that avoiding critical structures will most likely lead to inaccurate targeting.

The current study evaluated the steering capabilities of the needle in a gel phantom and in a tissue phantom. Neither of the phantoms contained critical structures that need to be avoided. This simplifies navigation thoroughly compared to an in vivo situation. The controllability of the needle is affected by the heterogeneity of tissue. The large heterogeneity of real tissue causes needles to deflect more, which stresses the need for active control of the needle trajectory. Besides this, the needle was controlled by one of the researchers, while one can expect more dexterity from an experienced physician, who could simultaneously insert the needle, control the tip direction, and verify the resulting trajectory on the continuously updated MR images. Extensive testing is required to gain insight in these factors. In such experiments, target accuracy, the number of attempts, and duration of the intervention should be measured to quantify the added value of this technology.

An important challenge for implementation in practice will be the selection of the imaging planes or volumes. In some cases, the imaging strategy as described here may be sufficient. In other cases, more sophisticated selection of imaging planes may be necessary, for example when the trajectory is expected or planned to be curved. Automatic selection of imaging plane could be guided by real-time tracking of the needle. This can be accomplished through shape sensing by means of fiber Bragg gratings that are integrated in the instrument, which is work in progress [9, 10]. In this case, the imaging planes of the MRI can be matched real-time with the position and orientation of the needle and its tip as measured with the fiber Bragg gratings. However, from a safety perspective, visualization of the tip on MRI with respect to the target area will remain indispensable to assure that the tip is at the intended location when the treatment is started. Therefore, further research should also focus on optimization and validation of needle tip visualization using MRI.

## Conclusion

A manually controlled steerable needle has been developed successfully. This needle provides active control of the needle trajectory and may increase the accuracy of needle positioning. Steering is initiated by adjusting the orientation of the needle tip. The needle was demonstrated to be compatible with MRI. The image quality in the direct vicinity of the needle was not affected, while the needle and its tip could be visualized clearly.
